# Anti‐seizure medications in patients with post‐stroke epilepsy: A survival analysis study

**DOI:** 10.1111/epi.18706

**Published:** 2025-11-07

**Authors:** Ippazio Cosimo Antonazzo, Carla Fornari, Gabriele Buongarzone, Pietro Ferrara, Giacomo Crotti, Alberto Zucchi, Paolo Angelo Cortesi, Davide Rozza, Lorenzo Giovanni Mantovani, Giampiero Mazzaglia

**Affiliations:** ^1^ Research Centre on Public Health (CESP) University of Milano‐Bicocca Monza Italy; ^2^ Epidemiology Unit, Bergamo Health Protection Agency Bergamo Italy; ^3^ Laboratory of Public Health, IRCCS Istituto Auxologico Italiano Milan Italy; ^4^ Department of Environmental and Prevention Sciences, University of Ferrara Ferrara Italy

**Keywords:** anti‐seizure medications, Italy, mortality, post‐stroke epilepsy, public health

## Abstract

**Objective:**

The role of antiseizure medications (ASMs) in patients with post‐stroke epilepsy (PSE) is still debated. Although a few studies have compared the efficacy of different ASMs on mortality in patients with PSE, overall evidence on the impact of ASM use on survival is limited. This study aimed to evaluate the association between ASM use and all‐cause mortality in patients with PSE.

**Methods:**

A cohort study was conducted using health care administrative database of Health Protection Agency of Bergamo (Italy). Individuals with a diagnosis of stroke followed by epilepsy onset between January 1, 2014 and December 31, 2017 were included. The date of epilepsy was considered as Index date (ID). Patients were followed from the ID until death, disenrollment, or end of follow‐up, whichever occurred first. Exposure to ASMs was defined as at least one dispensing within 30 days of the ID; patients without ASM dispensing during this period were considered non‐exposed. All‐cause mortality was analyzed using Cox proportional hazards models, with non‐exposure as the reference. Two analytical approaches were adopted: an intention‐to‐treat analysis and a time‐dependent analysis.

**Results:**

A total of 145 patients met the inclusion criteria: 107 ASM users and 38 non‐users. In the intention‐to‐treat analysis, ASM use was associated with a lower risk of all‐cause mortality (adjusted hazard ratio [HR]: 0.56; 95% confidence interval [CI]: 0.33–0.95). Consistent findings were observed in the time‐dependent analysis (adjusted HR: 0.39; 95% CI: 0.23–0.65). The sensitivity analyses confirmed the robustness of the results.

**Significance:**

In this population‐based cohort study, ASM use in patients with PSE was associated with a significantly reduced risk of all‐cause mortality compared to non‐use. These findings support the hypothesis that ASM treatment might be associated with positive effect in this high‐risk population.

**Key points**

- The use of antiseizure medications (ASMs) in patients with post‐stroke epilepsy (PSE) remains a matter of debate.
- Findings reveal a positive association between the use of ASMs and survival in patients with PSE.
- Future studies should be conducted to assess potential differences in ASM effects across different stroke subgroups.

## 1 INTRODUCTION

Stroke is a common cause of seizures and epilepsy in adults, accounting for ~11% of epilepsy diagnoses^1^ and 30%–55% of newly diagnosed seizures among older individuals.^2^, ^3^, ^4^, ^5^ The reported incidence of post‐stroke epilepsy (PSE) ranges from 2% to 20%, varying according to stroke subtype, cortical involvement, stroke severity, large stroke volume, stroke treatment (i.e., decompressive craniectomy, craniotomy, intravenous alteplase, or endovascular treatment), and seizure‐onset timing.^5^, ^6^, ^7^, ^8^, ^9^ The epidemiological data on PSE remain heterogeneous due to variations in its definition over time. Traditionally, PSE has been categorized into early and late epilepsy based on seizure onset following stroke. More recently, the International League Against Epilepsy (ILAE) redefined PSE as any seizure occurring seven or more days after a stroke,^10^, ^11^, ^12^, ^13^ whereas acute symptomatic seizures if they occur within 7 days of cerebrovascular disease, central nervous system infection, intracranial surgery, and traumatic brain injury.^12^, ^13^

Previous studies have indicated that PSE is associated with increased mortality risk and poorer functional outcomes, greater disability, and increased risk of dementia, compared with stroke patients without PSE.^11^, ^14^, ^15^, ^16^, ^17^, ^18^, ^19^ Timely identification and optimal management of seizures and epilepsy following stroke are therefore crucial for improving patient outcomes. Despite existing evidence highlighting the association between PSE and adverse clinical outcomes, there are no established clinical guidelines addressing the key aspects of PSE management,^20^ particularly: (1) whether all patients with PSE should receive treatment and the optimal timing of initiation, and (2) the most appropriate antiseizure medication (ASM) for PSE management.

Patients with epilepsy after brain insult such as stroke, central nervous system infection, or trauma have a high risk of a second unprovoked seizure (~60%).^10^ However, conflicting evidence exists regarding the efficacy of ASMs in both early and late seizures, suggesting that treatment decisions remain influenced by seizure characteristics and individual patient profiles rather than a standardized evidence‐based approach.

Furthermore, although several studies have examined ASM efficacy in patients with PSE, limited data exist regarding their impact on stroke recurrence and mortality. A recent study analyzing PSE patients treated with at least one ASM reported an increased mortality risk in individuals receiving phenytoin compared to those treated with newer‐generation ASMs over a 5‐year follow‐up period (hazard ratio [HR]: 1.64; 95% confidence interval [CI]: 1.06–2.55).^21^ Conversely, no significant differences in cardiovascular event occurrence were observed among different treatment groups.^21^

The selection of an appropriate ASM in PSE patients differs from that in patients with epilepsy alone. Patients with PSE are generally older, more likely to have multiple comorbidities, and often receive polypharmacy, thereby increasing their susceptibility to adverse drug effects. Consequently, ASM choice must consider factors such as potential side effects, tolerability, impact on rehabilitation and recovery, and drug–drug interactions.^20^

Currently, several ASMs are available. According to ILAE recommendations, carbamazepine, levetiracetam, phenytoin, and zonisamide may be beneficial for adults with focal seizures, whereas gabapentin and lamotrigine are preferred for elderly patients.^22^ Existing data suggest that over 70% of patients with PSE respond positively to ASM treatment,^17^, ^23^, ^24^, ^25^, ^26^, ^27^ although no significant differences in efficacy have been observed among most ASMs.

To the best of our knowledge, data on the association between ASM use in patients with PSE and mortality risk are limited.^21^, ^28^ For this reason, this study aimed at assessing the association between ASM use and all‐cause mortality in patients with PSE.

## 2 METHODS

### 2.1 Data sources

A cohort study was conducted using data from the health care administrative databases (HADs) of the local Health Protection Agency (HPA) of Bergamo (HPA‐Bergamo) in Italy. This database includes comprehensive health‐related information on ~1 million residents within the province of Bergamo and has been employed previously in epidemiological and pharmacoepidemiological research. For this study, the following databases were linked: (1) the population registry, which records dates and reasons for entry into and exit from the HPA catchment area; (2) the hospital discharge database (HDD), which contains information on hospital admission and discharge, including dates and diagnoses coded according to the International Classification of Diseases, Ninth Revision, Clinical Modification (ICD‐9‐CM); (3) the emergency department (ED) database, which includes dates of ED access and diagnosis coded according to the ICD‐9‐CM; and (4) the drug dispensing registry, which includes information on dispensing date, doses, and number of dispensed drugs through community or hospital pharmacies. This study was approved by the “Comitato Etico Territoriale Lombardia 6” of the Lombardy Region, with number 0038081/24, approved on June 18th, 2024.

### 2.2 Study population

The study population included all inhabitants ≥45 years of age who received a diagnosis of ischemic or hemorrhagic stroke between January 1, 2014 and December 31, 2017, followed by an ED access or hospitalization for epilepsy (ICD9‐CM: 345) between 7 days and 2 years from the stroke event (ICD‐9‐CM: 430–432, 433.x1, 434.x1, and 436). The date of the first PSE diagnosis was considered as the index date (ID). Individuals were excluded if they had less than 2 years of available data prior to the ID; concomitant diagnosis of ischemic and haemorrhagic stroke, brain injury, and rehabilitation care within 30 days prior the ID; ASM use within 180 days prior to the date of hospitalization for stroke; a diagnosis of epilepsy within 2 years prior to the ID; or a diagnosis of malignancy within the 2 years prior to the ID.

### 2.3 Exposure

Two types of analyses were performed in this study: an intention‐to‐treat (ITT) analysis and a time‐dependent analysis. In the ITT approach, patients were classified into two mutually exclusive groups based on ASM exposure. Individuals were considered exposed if they received at least one dispensing of an ASM within 30 days after the index date; those with no dispensing during this period were classified as non‐exposed.

For the time‐dependent analysis, treatment episodes were constructed to capture periods of uninterrupted ASM use during follow‐up. The duration of each dispensing was estimated by dividing the total amount of active substance by the corresponding defined daily dose (DDD). A grace period of 40 days was allowed between the end of drug coverage and the next dispensing to account for potential delays in medication renewal. If a new dispensing occurred within the coverage window of an existing episode, any overlapping day was added to extend the end date of the episode. Treatment was considered discontinued at the end of drug coverage of the last dispensing when no further refill occurred within the grace period. Patients were classified as exposed to ASMs during treatment episodes, and as unexposed during intervals outside these episodes.

### 2.4 Outcome

The primary outcome was all‐cause mortality occurring during follow‐up. Mortality data were obtained from the HPA's databases.

### 2.5 Follow‐up

Each individual contributed person‐time from the ID until the earliest occurrence of one of the following events: (1) death (study outcome), (2) disenrollment from the health care system, or (3) end of the follow‐up period (5 years after the ID).

### 2.6 Patients’ covariates

For each patient, demographic variables including sex and age were recorded at the ID. Clinical information—specifically comorbidities and concomitant medication use—was assessed during the period preceding the ID. The number of distinct concomitant drug therapies (based on Anatomical Therapeutic Chemical (ATC) classification 5th‐Level codes) dispensed within 6 months before the ID was calculated. Comorbidities were identified using diagnostic codes recorded within the 2 years prior to the ID. The following comorbidities were considered: hypertension, diabetes, dyslipidaemia, atrial fibrillation, ischemic heart disease, heart failure, mood and anxiety disorders, chronic pulmonary disease, migraine, chronic kidney disease, moderate–severe liver disease, and chronic disease of pancreas and intestine. The Congestive heart failure, Hypertension, Age (>=75 years, double), Diabetes, Stroke/TIA (double), Vascular disease, Age (65–74 years) and Sex Category (female) (CHA_2_DS_2_‐VASc) score and Charlson Comorbidity Index (CCI) were also computed at baseline.^29^, ^30^, ^31^ Furthermore, during the study period the proportion of days covered (PDCs) with ASM were estimated.^32^ Individuals were classified into three mutually exclusive categories: high adherence (PDC ≥80), intermediate adherence (PDC 40–79), and low adherence (PDC ≤39).

### 2.7 Data analysis

Descriptive statistics were reported as number and percentage for categorical variables and as mean (SD) for continuous variables. Differences between the two groups were examined using the Pearson's chi‐square or Fisher exact test for categorical variables, and non‐parametric Wilcoxon test for continuous variables.

The association between ASM use and mortality during follow‐up was assessed by using univariate Cox regression analysis to identify predictors, followed by stepwise multivariate regression to select significant variables. Cox‐proportional hazard model assumption was checked using Schoenfeld residuals. Results were expressed as hazard ratio (or HR) with 95% confidence intervals (or 95% CIs).

To account for the potential different use of ASMs (different therapeutic regimens) in post‐stroke individuals, a sensitivity analysis was performed by adopting a grace period of 60 days. In addition, to account for potential factors influencing patients' survival during the first period after PSE, two sensitivity analyses were conducted including only individuals with at least 1 month and 3 months of follow‐up in the time‐dependent analysis. Furthermore, to account for potential confounding by indication of treatment, an inverse probability of treatment weighting (IPTW) analysis was performed. IPTW analysis uses the propensity score to balance baseline patient characteristics in ASM users and non‐users by weighting each individual by the inverse probability of receiving the actual treatment.^33^ The propensity score was estimated using a logistic regression model, including the following predictors: age, sex, type of stroke, time at epilepsy onset, CHA_2_DS_2_VASc, CCI, number of comorbidities, and number of concomitant therapies. We adopted stabilized weights to reduce the effect of extreme weights. Graphical inspection of propensity score distribution pre‐ and post‐ IPTW was assessed (FigureA1). The balance of baseline covariates between treatment groups was assessed by standardized differences, using a threshold of 0.1 to indicate imbalance. Covariates that had a standardized difference among 0.1–0.5 were inserted as predictors in Cox regression models also after IPTW.

All statistical analyses were performed using R 4.0.5 (R Foundation for Statistical Computing, Vienna, Austria) and SAS 9.4 (SAS Institute, Cary, North Carolina, USA).

## 3 RESULTS

**FIGURE 1:**
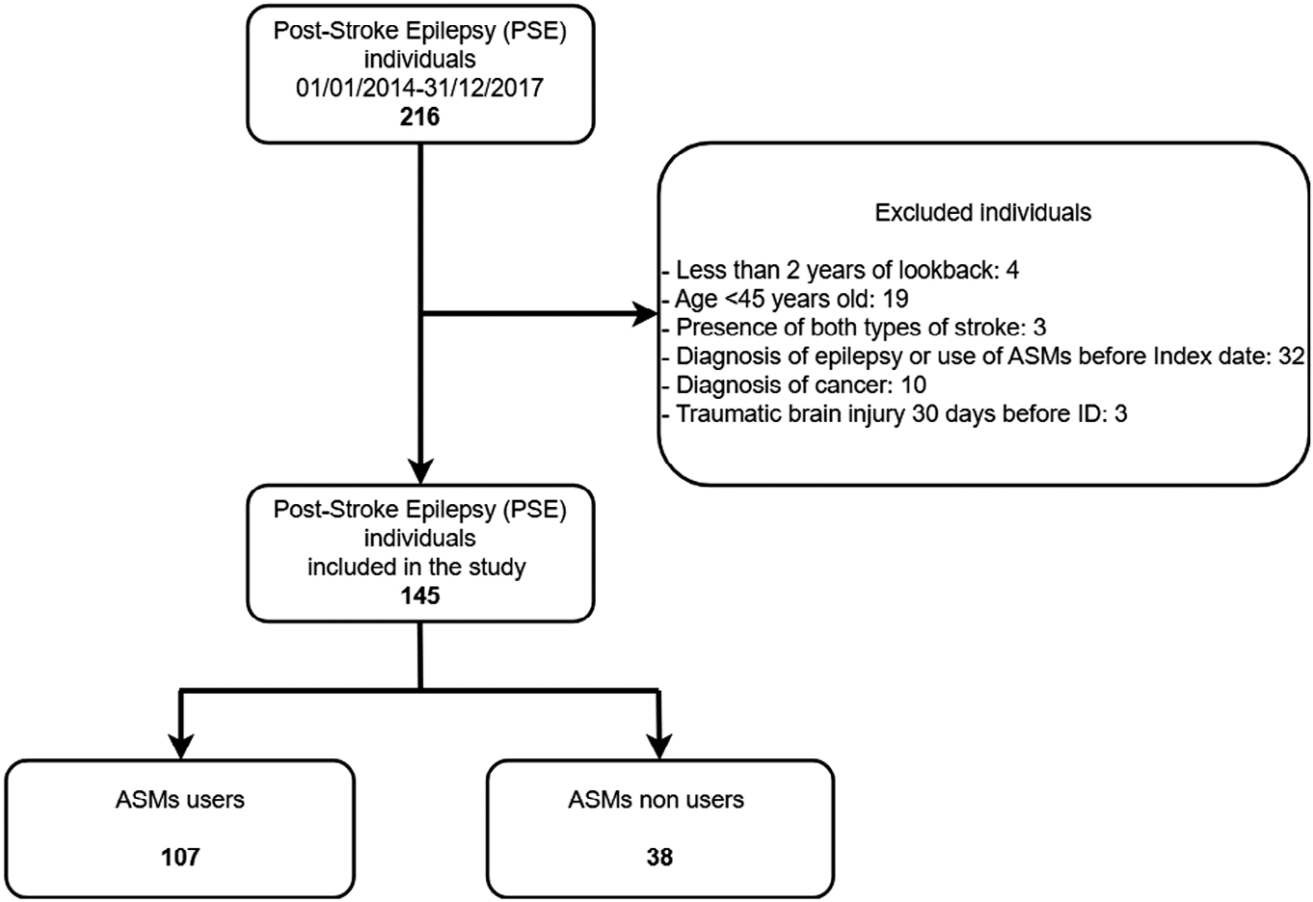
Flowchart of study cohort selection. ASMs, antiseizure medications.

During the study period (January 1, 2014, to December 31, 2017), 216 subjects experienced PSE in the HPA‐Bergamo area. After exclusion criteria were applied, 145 individuals were included in the analyses. Figure 1 illustrates the inclusion and exclusion process used to identify the final cohort of patients with PSE from the HAD of the HPA‐Bergamo area.

Table 1 presents the baseline characteristics of ASM users and non‐users. The mean time between stroke and the first epileptic event was 8.90 months (SD: 5.57) in the ASM user group and 6.47 months (SD: 5.96) in the non‐user group (*p*‐value < 0.05). The mean follow‐up was 45.59 months (SD: 19.54) for ASM users and 29.43 (SD: 25.74) for non–users (*p*‐value < 0.05). In both groups, the majority of patients were male (57% in ASM users and 55% in non‐users). The mean age was 70 years (SD: 11.6) for ASM users and 75 years (SD: 11.7) for non‐users (*p*‐value < 0.05). No statistically significant difference was observed in the mean number of comorbidities among the two study groups: 1.70 (SD: 0.95) in ASM users and 1.97 (SD: 1.26) in non‐users. Similarly, no significant differences were observed in CCI and CHA_2_DS_2_‐VASc score.

**TABLE 1:** Demographic and clinical characteristics of ASM users and non‐users.

	ASM non‐users	ASM users	All
Total number	38	107	145
Ischemic stroke, *n* (%)	26 (68.4)	82 (76.6)	108 (74.5)
Time at epilepsy onset, months,			
mean ± SD	6.47 ± 5.96	8.90 ± 5.57*	8.26 ± 5.76
Sex, *n* (%)			
Men	21 (55.3)	61 (57.0)	82 (56.6)
Age, years, mean			
± SD	74.7 ± 11.7	70.4 ± 11.6*	71.5 ± 11.8
Number of hospitalizations in the pre‐index period, *n* (%)			
0	10 (26.3)	26 (24.3)	36 (24.8)
1	11 (29.0)	31 (28.9)	42 (29.0)
2	8 (21.0)	25 (23.4)	33 (22.8)
≥3	9 (23.7)	25 (23.4)	24 (23.5)
Mean ± SD	1.6 ± 1.5	1.6 ± 1.3	1.6 ± 1.4
Number of comorbidities, *n* (%)			
1	17 (44.7%)	52 (48.6%)*	69 (39.3%)
2	14 (36.8%)	42 (29.0%)*	56 (38.6%)
3	1 (2.6%)	10 (9.3%)*	11 (7.6%)
≥4	6 (15.8%)	3 (2.8%)*	9 (6.2%)
mean ± SD	1.97 ± 1.26	1.70 ± 0.95	1.77 ± 1.05
Charlson Comorbidity Index,			
mean ± SD	2.21 ± 1.51	2.09 ± 1.36	2.12 ± 1.40
CHA_2_DS_2_‐VASc,			
mean ± SD	5.47 ± 1.61	4.95 ± 1.57	5.09 ± 1.59
Number of drug therapies at index event, *n* (%)			
0	5 (13.2%)	5 (4.7%)	10 (6.9%)
1	3 (7.9%)	7 (6.5%)	10 (5.9%)
2	6 (15.8%)	8 (7.5%)	14 (6.7%)
3	6 (15.8%)	12 (11.2%)	18 (12.4%)
≥4	18 (47.4%)	75 (70.0%)	93 (64.1%)
mean ± SD	3.47 ± 2.27	4.31 ± 1.97*	4.09 ± 2.08
Follow‐up time, months,			
mean ± SD	29.43 ± 25.74	45.59 ± 19.54*	41.35 ± 22.41

Abbreviation: ASM, antioseizure medication; CHA2DS2‐VASc, Congestive heart failure, Hypertension, Age (>=75 years, double), Diabetes, Stroke/TIA (double), Vascular disease, Age (65‐74 years) and Sex Category (female); PDC, proportion of days covered; NIHSS, National Institutes of Health Stroke Scale; SD, standard Deviation.

* *p*‐value < 0.05.

Among ASM users, the most frequently used drug was levetiracetam (76.6%), followed by valproic acid (10.3%), oxabarzepine (2.80%), phenobarbital (1.87%), lamotrigine (1.87%), zonisamide (1.87%), lacosamide (1.87%), and other with less than 1% of use. Furthermore, in the study period, among treated individuals, 53% had a PDC ≥80%, 28% between 40% and 79%, and 10% less than 40%.

**FIGURE 2:**
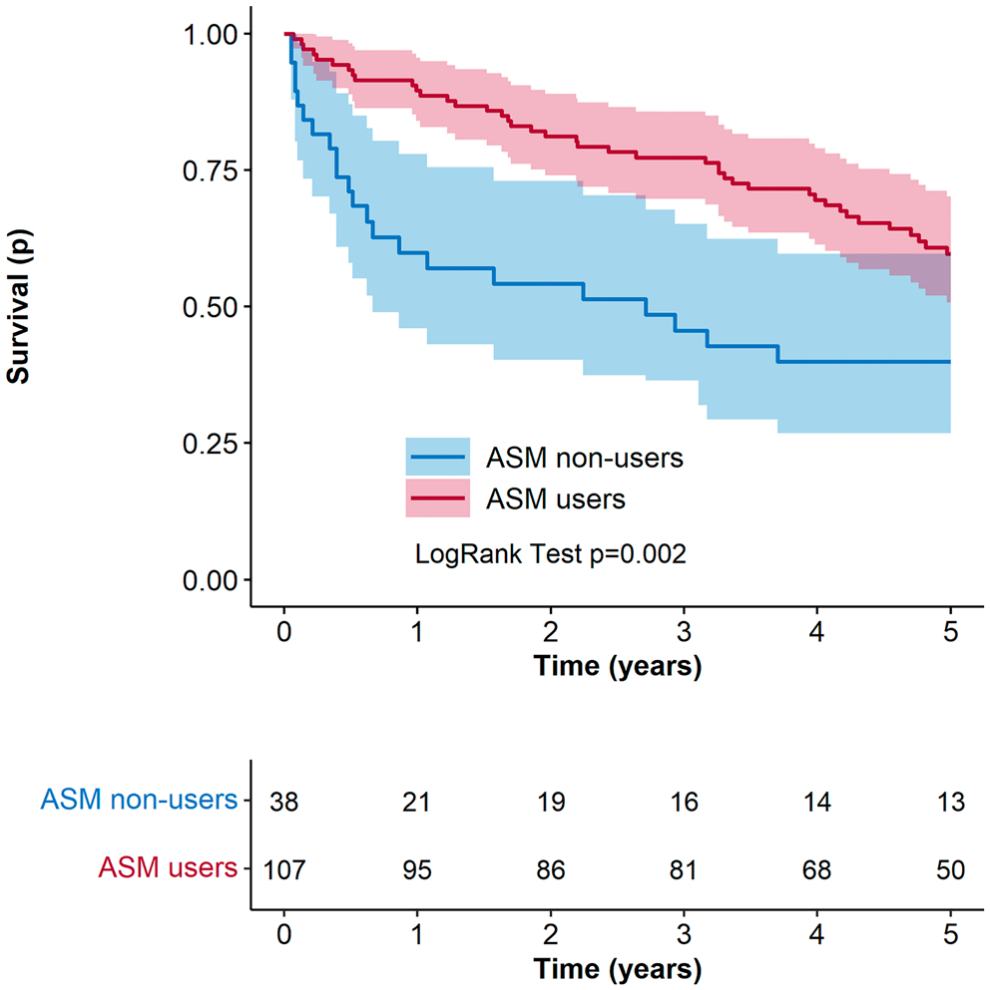
Kaplan–Meier survival curves for all‐cause mortality in patients with post‐stroke epilepsy, stratified by antiseizure medication (ASM) use.

During the study period, a total of 63 deaths were recorded. Figure 2 presents the crude survival curves estimated using the Kaplan–Meier method, stratified by ASM exposure status. A statistically significant difference in overall survival was observed between ASM users and non‐users (*p* < 0.05), with ASM users showing higher survival probability over time.

Table 2 presents the results of both univariate and multivariate Cox regression analyses conducted under the ITT and time‐dependent approaches after checking and confirming that the proportional hazard was not violated. In the ITT analysis, ASM use was associated with a significantly lower risk of all‐cause mortality compared to non‐use (HR: 0.46; 95% CI: 0.27–0.78). Older age (HR: 1.09; 95% CI: 1.06–1.12), higher CHA_2_DS_2_‐VASc score (HR: 1.51; 95% CI: 1.28–1.77), and a higher number of comorbidities (HR: 1.40; 95% CI: 1.13–1.73) were significantly associated with increased mortality. In the multivariate ITT model, ASM use remained significantly associated with a reduced risk of death (adjusted HR: 0.56; 95% CI: 0.33–0.95), whereas age (HR: 1.09; 95% CI: 1.06–1.13) and number of comorbidities (HR: 1.42; 95% CI: 1.14–1.77) remained independent predictors of increased mortality.

**TABLE 2:** Association between antiseizure medication (ASM) use and all‐cause mortality in patients with post‐stroke epilepsy.

	Intention‐to‐treat analysis		Time‐dependent analysis	
	Univariate	Multivariate	Univariate	Multivariate
	Hazard ratio (95% CI)	Hazard ratio (95% CI)	Hazard ratio (95% CI)	Hazard ratio (95% CI)
ASM users (ref. non‐users)	**0.46 (0.27–0.77)**	**0.56 (0.33–0.95)**	**0.37 (0.23–0.61)**	**0.39 (0.23–0.65)**
Age	**1.09 (1.06–1.12)**	**1.09 (1.06–1.13)**	**1.09 (1.06–1.12)**	**1.09 (1.06–1.13)**
Sex (ref. men)	0.81 (0.49–1.32)		0.79 (0.48–1.30)	
Hemorrhagic stroke (ref. ischemic)	1.28 (0.71–2.33)		1.37 (0.74–2.53)	
CHA_2_DS_2_‐VASc	**1.51 (1.28–1.77)**		**1.55 (1.32–1.82)**	
No. of comorbidities	**1.40 (1.13–1.73)**	**1.42 (1.14–1.77)**	**1.46 (1.17–1.81)**	**1.61 (1.3–1.99)**
Charlson Comorbidity Index	1.16 (0.98–1.37)		**1.2 (1.02–1.42)**	
No. of hospitalization	0.97 (0.80–1.17)		0.95 (0.79–1.15)	
No. of concomitant therapies	1.09 (0.95–1.24)		1.11 (0.97–1.26)	
Time at epilepsy onset (months)	1.00 (0.95–1.05)		1.01 (0.96–1.06)	
PDC (ref. 0–39)			–	
40–79	0.61 (0.32–1.15)		–	
≥80	0.50 (0.28–0.88)		–	

*Note*: Bold values are significant (*p* < 0.05).

Abbreviations: 95% CI, 95% confidence intervals; CHA2DS2‐VASc, Congestive heart failure, Hypertension, Age (>=75 years, double), Diabetes, Stroke/TIA (double), Vascular disease, Age (65–74 years) and Sex Category (female); PDC, proportion of days covered.

Findings from the time‐dependent analysis were consistent with the ITT results. In the univariate model, ASM use was associated with a lower risk of death (HR: 0.37; 95% CI: 0.23–0.61), whereas age (HR: 1.09; 95% CI: 1.06–1.12), CHA_2_DS_2_‐VASc score (HR: 1.55; 95% CI: 1.32–1.82), number of comorbidities (HR: 1.46; 95% CI: 1.17–1.81), and CCI (HR: 1.20; 95% CI: 1.02–1.42) were significantly associated with increased mortality. In the multivariate time‐dependent model, ASM use continued to show a significant negative association (HR: 0.39; 95% CI: 0.23–0.65), whereas age (HR: 1.09; 95% CI: 1.06–1.13) and number of comorbidities (HR: 1.61; 95% CI: 1.30–1.99) showed a positive association with the study outcome.

Table S1 reports the results of a sensitivity analysis conducted using a 60‐day grace period in the time‐dependent exposure model. The findings were consistent with those of the primary analysis. In the multivariate model, ASM use remained significantly associated with a lower risk of all‐cause mortality (HR: 0.35; 95% CI: 0.21–0.59) and older age (HR: 1.09; 95% CI: 1.06–1.13), and a higher number of comorbidities (HR: 1.61; 95% CI: 1.30–1.99) continued to predict increased mortality risk. As reported in Table A2 in the supplementary material, sensitivity analyses including individuals with at least 1 month and 3 months of follow‐up showed similar results. Among patients with at least 1 month of follow‐up, in the time‐dependent multivariate model, the use of ASMs was negatively associated with all‐cause mortality (HR: 0.39; 95% CI: 0.23–0.66), whereas older age (HR: 1.09; 95% CI: 1.06–1.13), and higher number of comorbidities (HR: 1.59; 95% CI: 1.28–1.98) were positively associated with an increased risk of all‐cause mortality. Similar results were observed in the time‐dependent multivariable analysis restricted to patients with at least 3 months of follow‐up: ASM use (HR: 0.41; 95% CI: 0.23–0.72), age (HR: 1.08; 95% CI: 1.04–1.11), and number of comorbidities (HR: 1.53; 95% CI: 1.20–1.96).

Tables A3 and A4 and Figures A1 and A2 (supplementary material) report the results of a sensitivity analysis conducted using the IPTW methodology. Demographic and clinical characteristics of ASM users and ASM non‐users were balanced after applying IPTW (Table A3 and Figure A2). In fact, as reported in Figure A2, after IPTW, analyzed covariates were balanced with SD between −0.1 and 0.1 apart for age, number of comorbidities, and CCI, which showed a SD between −0.5 and 0.5. These covariates, apart from the CCI due to its high correlation with the number of comorbidities, were included in the adjusted model as reported in Table A4 in the supplementary material. In the ITT, the factors associated with increased mortality were age (HR: 1.09; 95% CI: 1.06–1.13) and the number of comorbidities HR (1.36; 95% CI: 1.06–1.73); the use of ASM showed a non‐significant association with mortality risk (HR: 0.62; 95% CI: 0.35–1.09) (Table A4). The time‐dependent analysis showed a significant negative association between the use of ASMs and mortality (HR: 0.40; 95% CI: 0.24–0.76), and a positive association between age (HR: 1.09; 95% CI: 1.06–1.13) and number of comorbidities (HR: 1.60; 95% CI: 1.28–1.99) and mortality (Table A4).

## 4 DISCUSSION

This population‐based study cohort demonstrated that the use of ASMs in patients with PSE is associated with a significantly lower risk of all‐cause mortality compared to non‐use. Both the ITT and time‐dependent analyses yielded consistent results, suggesting a positive association between ASM use and decreased mortality risk in this high‐risk population, which should be confirmed by additional studies. Notably, the reduction in mortality risk exceeded 50% in both analytic approaches. Sensitivity analyses, especially time‐dependent analysis, corroborated findings from the main analysis.

To our knowledge, this is one of the first studies to specifically investigate the impact of ASM use on overall survival in a real‐world PSE cohort. Previous studies have focused primarily on the comparative effectiveness of different ASMs on stroke recurrence and mortality in patients with PSE. For instance, a Swedish study found that lamotrigine, followed by levetiracetam, oxcarbazepine, and carbazepine, was associated with the highest 3‐year survival rates. Furthermore, lamotrigine was associated with significantly lower mortality compared to carbamazepine, whereas valproic acid was associated with an increased risk of death.^28^ Similarly, a study utilizing data from the National Health Insurance Research Database (NHIRD) found that phenytoin was associated with increased mortality risk over a 5‐year follow‐up compared to newer ASMs such as clobazam, gabapentin, lacosamide, lamotrigine, levetiracetam, oxcarbazepine, perampanel, pregabalin, rufinamide, tiagabine, topiramate, vigabatrin, and zonisamide.

Our findings complement these studies by showing that, regardless of ASM class, their use may be beneficial in reducing mortality—particularly since most patients (76%) in our cohort received levetiracetam, which has a favorable pharmacological profile and is well tolerated in older populations, allowing us to cautiously speculate that this medication may be associated with improved survival outcomes.

Several mechanisms might explain the observed association. First, untreated or poorly controlled epilepsy is associated with an increased risk of adverse events including sudden unexpected death in epilepsy, falls, trauma, and aspiration pneumonia.^34^, ^35^ Second, PSE may itself be a marker of more severe brain injury or higher stroke burden,^36^, ^37^ and effective seizure management may reduce neurological complications, improve rehabilitation participation, and mitigate the cascading risks associated with recurrent seizures. In addition, improved adherence to medical follow‐up and care among ASM users could reflect an overall better health management,^38^ introducing potential healthy adherer bias.

Stroke remains one of the leading causes of disability and mortality worldwide, and epilepsy is a well‐recognized complication following both ischemic and hemorrhagic strokes. Despite its prevalence and clinical relevance, treatment recommendations for patients with PSE remain limited. These patients represent a clinically relevant subgroup warranting further research, particularly regarding the impact of ASMs on long‐term survival. Notably, some ASMs, especially older agents, have been associated with increased cardiovascular risk. Although data on ASM use in patients with PSE specifically are scarce, prior studies in epilepsy populations have shown that older ASMs are linked to higher risk of myocardial infarction, stroke, cardiovascular death, and all‐cause death.^39^, ^40^ Moreover, poor epilepsy control has itself been associated with increased mortality.^19^, ^41^ Although the underlying mechanisms by which PSE influences mortality rates are not fully understood, and the role of ASMs in this pathway remains to be clarified, continued research in this area is crucial for guiding clinical decisions and optimizing treatment strategies for patients with PSE.

Our findings have relevant implications for clinical practice and public health. Despite the absence of specific recommendations regarding treatment initiation (and choice) of ASM for PSE management,^42^ our results support a potential proactive use of ASM in this patient group. Given the aging population and the growing burden of both stroke and epilepsy, identifying strategies that reduce mortality is essential. ASMs, when appropriately selected and monitored, may represent an effective intervention to improve long‐term outcomes in these patients.

The main strength of our study is the use of a large population‐based administrative database with complete coverage of hospitalizations, emergency care, and drug dispensing, within the Italian health care service context. Furthermore, in Italy, ASMs are reimbursed by the National Healthcare Service and can be dispensed by pharmacies to the general population only with a medical prescription. This enhances the accuracy of medication data. In addition, the study benefits from long follow‐up of patients that allowed assessment of the long‐term effects of ASMs use on the study outcome.

Nevertheless, the findings of this study should also be interpreted with caution in light of some limitations. First, the lack of clinical information within the HAD, such as stroke severity (e.g., National Institutes of Health Stroke Scale NIHSS), epilepsy type, lesion location and characteristics, epilepsy semiology, or functional status limits adjustment for important confounders that can influence ASMs prescription and mortality of patients. Second, although we adjusted the main analysis for several factors and performed several sensitivity analyses (i.e., by considering longer grace period, by including only individuals with at least 1 month and 3 months of follow‐up, and by using the IPTW analysis), residual confounding from unmeasured factors (i.e., smoking, alcohol use, socioeconomic status) cannot be excluded. Third, medication adherence was inferred from the dispensing data, which may not reflect actual use in terms of actual drug intake. Furthermore, although the PDC is a well‐established method for estimating medication adherence in (pharmaco)epidemiological studies, it does not capture qualitative aspects such as dosing accuracy and timing, which might influence treatment effectiveness. Fourth, the treatment pattern of patients with epilepsy might be intricate. To account for potential differences in dose of therapies taken by patients, we adopted 40 days of grace period as well as performed a sensitivity analysis by using a longer grace period of 60 days. In addition, the lack of information in the health care database regarding drug indication or therapeutic regimen prevented assessment of whether treatment was consistent with guidelines recommendations. The absence of drug indication data did not allow inclusion of patients with stroke receiving ASMs who may have been diagnosed with epilepsy in the community setting. Future studies integrating multiple data sources (i.e., general practioner databases) could help to clarify the role of ASMs in patients with PSE who are diagnosed and managed in community setting. Moreover, information on cause of death was not available in the database, which precludes determining whether mortality was related to uncontrolled seizures. Although the aim of the study was to assess all‐cause mortality, future studies should be designed specifically to investigate the association between ASM use in PSE individuals and seizure‐related mortality. Finally, the relatively small sample size limited subgroup analyses, including comparison among different ASM classes, as well as assessed the association between ASM use and occurrence of new stroke event.

Further research is needed to confirm our findings in larger and more diverse populations, ideally integrating clinical datasets with more granular stroke and epilepsy characteristics. Comparative effectiveness studies between different ASMs, including head‐to‐head randomized trials, are also warranted to identify the optimal pharmacological approach. Finally, research should explore whether the potential benefits of ASM use vary according to stroke subtype, timing of seizure onset, or severity of epilepsy.

## 5 CONCLUSION

This observational study based on a health care database revealed a significant difference in survival between patients with PSE who were treated with ASMs and those who were not. Specifically, ASM use was associated with a substantial reduced mortality risk. These findings suggest the potential for use of ASMs in patients with PSE. Given the variety of available antiseizure therapies and the heterogenicity of clinical presentation, further observational studies and randomized controlled trials are warranted to confirm the potential positive effect of ASMs in this population and to determine the most effective ASM regimens for patients with PSE. However, given the number of confounding factors that cannot be fully accounted for in observational studies, treatment decisions should be guided by clinicians based on patients' individual characteristics. Future research should also explore whether treatment effects differ according to stroke subtype and investigate the potential impact of this factor on mortality risk.

## AUTHOR CONTRIBUTIONS

Conceptualization: I.C.A. and C.F. Methodology: I.C.A., C.F., and G.C. Data curation: C.F., D.R., and G.B Writing – original draft preparation: I.C.A. and C.F. Writing – review and editing: all authors. All authors have read and agreed to the final version of the manuscript.

## FUNDING INFORMATION

This research received no external funding.

## CONFLICT OF INTEREST STATEMENT

I.C.A., C.F., G.B., P.F., G.C., A.Z., D.R., and G.M. have no disclosure to declare. P.A.C. has received a research grant from Baxalta, now part of Shire, and speaking honoraria from Pfizer and Roche. L.G.M. reported receiving grants from Bayer, Daiiki‐Sankyo and Boehringer Ingelheim outside the submitted work and speaker fees from Pfizer and Bayer.

## ETHICS STATEMENT

We confirm that we have read the Journal's position on issues involved in ethical publication and affirm that this report is consistent with those guidelines.

## INSTITUTIONAL REVIEW BOARD STATEMENT

This study was approved by the “Comitato Etico Territoriale Lombardia 6” of the Lombardy Region. Study protocol 0038081/24 on June 18, 2024.

## INFORMED CONSENT STATEMENT

Not applicable.

## Supporting information


Appendix S1.

## Data Availability

The data that support the findings of this study are available from the corresponding author upon reasonable request.
