# Supplementary material for: Anti‐seizure medications in patients with post‐stroke epilepsy: A survival analysis study
Source: Epilepsia. 2025 Nov 7;67(2):686–95. doi: 10.1111/epi.18706 (PMC12927701; doi:10.1111/epi.18706)

**Supplementary material**

**Title:** Anti-seizure medications in patients with post-stroke epilepsy: a survival analysis study.

**Authors**: Ippazio Cosimo Antonazzo^1,2,3,4^, Carla Fornari^1^, Gabriele Buongarzone^1^, Pietro Ferrara^1,3^, Giacomo Crotti^1,2^, Alberto Zucchi^2^, Paolo Angelo Cortesi^1,3^, Davide Rozza^1^, Lorenzo Giovanni Mantovani^1,3^, Giampiero Mazzaglia^1^

**Affiliation:**

1. Research Centre on Public Health (CESP), University of Milano-Bicocca, Monza, Italy.

2. Epidemiology Unit, Bergamo Health Protection Agency, Bergamo, Italy.

3. Laboratory of Public Health, IRCCS Istituto Auxologico Italiano, Milan, Italy.

4. Department of Environmental and Prevention Sciences, University of Ferrara, Ferrara, Italy.

**Corresponding author**

Carla Fornari, PhD

Research Centre on Public Health

University of Milano-Bicocca

Via Pergolesi 33

20900 Monza, Italy

Table A1. Association between antiseizure medication use and mortality in patients with post-stroke epilepsy, using a 60-days grace period in the time-dependent analysis.

|  | **Time-Dependent analysis** | |
| --- | --- | --- |
|  | **Univariate** | **Multivariate** |
|  | **Hazard Ratio (95%CI)** | **Hazard Ratio (95%CI)** |
| ASM users (ref. non-users) | **0.33 (0.20-0.54)** | **0.35 (0.21-0.59)** |
| Age | **1.09 (1.06-1.12)** | **1.09 (1.06-1.13)** |
| Sex (ref. men) | 0.79 (0.48-1.3) |  |
| Haemorrhagic stroke (ref. ischemic) | 1.37 (0.74-2.53) |  |
| CHA2DS2-VASc | **1.55 (1.32-1.82)** |  |
| N of comorbidities | **1.46 (1.17-1.81)** | **1.61 (1.30-1.99)** |
| Charlson Index | 1.20 (1.02-1.42) |  |
| N of hospitalization | 0.95 (0.79-1.15) |  |
| N of concomitant therapies | 1.11 (0.97-1.26) |  |
| Time at epilepsy onset (months) | 1.01 (0.96-1.06) |  |

*95%CI= 95% confidence intervals*

Table A2. Association between antiseizure medication use and mortality in patients with post-stroke epilepsy. Time dependent analysis performed among individuals with at least 1 month and 3 months of follow-up.

|  | **Analysis restricted among individuals with at least 1 month of follow-up** |  | **Analysis restricted among individuals with at least 3 months of follow-up** |
| --- | --- | --- | --- |
|  | **Hazard Ratio (95%CI)** |  | **Hazard Ratio (95%CI)** |
| ASM users (ref. non-users) | **0.39 (0.23-0.66)** |  | **0.41 (0.23-0.72)** |
| Age | **1.09 (1.06-1.13)** |  | **1.08 (1.04-1.11)** |
| N of comorbidities | **1.59 (1.28-1.98)** |  | **1.53 (1.20-1.96)** |

*95%CI= 95% confidence intervals*

Table A3. Demographic and clinical characteristics of ASM users and non-users (Inverse Probability of Treatment Weighting analysis).

|  | **ASM non- users** | **ASM users** | **All** |
| --- | --- | --- | --- |
| **Total number** | 37 | 106 | 143 |
| **Ischemic stroke - N (%)** | 27 (74.4) | 78 (73.0) | 105 (73.4) |
| **Time at epilepsy onset (months)** | | | |
| Mean ± standard deviation | 7.77 ± 5.8 | 8.90 ± 5.50 | 8.12 ± 5.57 |
| **Sex** |  |  |  |
| Men - n (%) | 21 (57.6) | 61(57.6) | 82 (57.6) |
| **Age** |  |  |  |
| Mean ± standard deviation | 72.9 ± 11.3 | 71.5 ± 11.3 | 71.9 ± 11.3 |
| **Number of hospitalizations in the pre-index period** | | | |
| 0 – n (%) | 11 (29.3) | 29 (27.7) | 40 (28.1) |
| 1 – n (%) | 10 (27.6) | 31 (29.4) | 41 (28.9) |
| 2 – n (%) | 9 (23.1) | 23 (21.5) | 32 (21.9) |
| ≥3 – n (%) | 7 (20.0) | 23 (21.4) | 30 (21.4) |
| Mean ± standard deviation | 1.4 ± 1.3 | 1.5 ± 1.3 | 1.5 ± 1.3 |
| **Number of comorbidities** | | | |
| 1 – n (%) | 20 (54.9%) | 52 (48.7%) | 72 (50.3%) |
| 2 – n (%) | 11 (29.8%) | 40 (38.0%) | 51 (35.8%) |
| 3 – n (%) | 3 (7.4%) | 11 (10.0%) | 14 (9.6%) |
| ≥4 – n (%) | 3 (7.9%) | 3 (3.0%) | 6 (4.2%) |
| Mean ± standard deviation | 1.7 ± 1.0 | 1.7 ± 1.0 | 1.7 ± 1.0 |
| **Charlson Index** | | | |
| Mean ± standard deviation | 1.9 ± 1.2 | 2.1 ± 1.4 | 2.1 ± 1.3 |
| **CHA₂DS₂-VASc** | | | |
| Mean ± standard deviation | 5.1 ± 1.5 | 5.0 ± 1.5 | 5.0 ± 1.5 |
| **Number of drug therapies at index event** | | | |
| 0 – n (%) | 4 (10.8%) | 7 (6.5%) | 11 (7.6%) |
| 1 – n (%) | 2 (4.9%) | 9 (8.3%) | 11 (7.4.%) |
| 2 – n (%) | 3 (8.7%) | 9 (8.4%) | 12 (8.5%) |
| 3 – n (%) | 6 (16.6%) | 12 (11.0%) | 18 (12.4%) |
| ≥4 – n (%) | 22 (59.0%) | 70 (65.8%) | 92 (65.7%) |
| Mean ± standard deviation | 4.0 ± 2.2 | 4.1 ± 2.1 | 4.1 ± 2.1 |
| **Follow- up time (months)** | | | |
| Mean ± standard deviation | 34.97 ± 25.75 | 45.43 ± 19.73 | 42.76 ± 21.86 |

Table A4. Association between antiseizure medication (ASM) use and all-cause mortality in patients with post-stroke epilepsy: multivariate analysis using the Inverse Probability of Treatment Weighting analysis.

|  | **Intention-To-Treat Analysis** | **Time-Dependent Analysis** |  |
| --- | --- | --- | --- |
|  | **Multivariate Hazard Ratio (95%CI)** | **Multivariate Hazard Ratio (95%CI)** |  |
| ASM users (ref. non-users) | 0.62 (0.35-1.09) | **0.40 (0.24-0.67)** |  |
| Age | **1.09 (1.06-1.13)** | **1.09 (1.06-1.13)** |  |
| N of comorbidities | **1.36 (1.06-1.73)** | **1.60 (1.28-1.99)** |  |

*95%CI= 95% confidence intervals*

Figure A1. Propensity score distribution in ASMs users and ASMs non-users pre- and post- Inverse Probability of Treatment Weighting (IPTW).


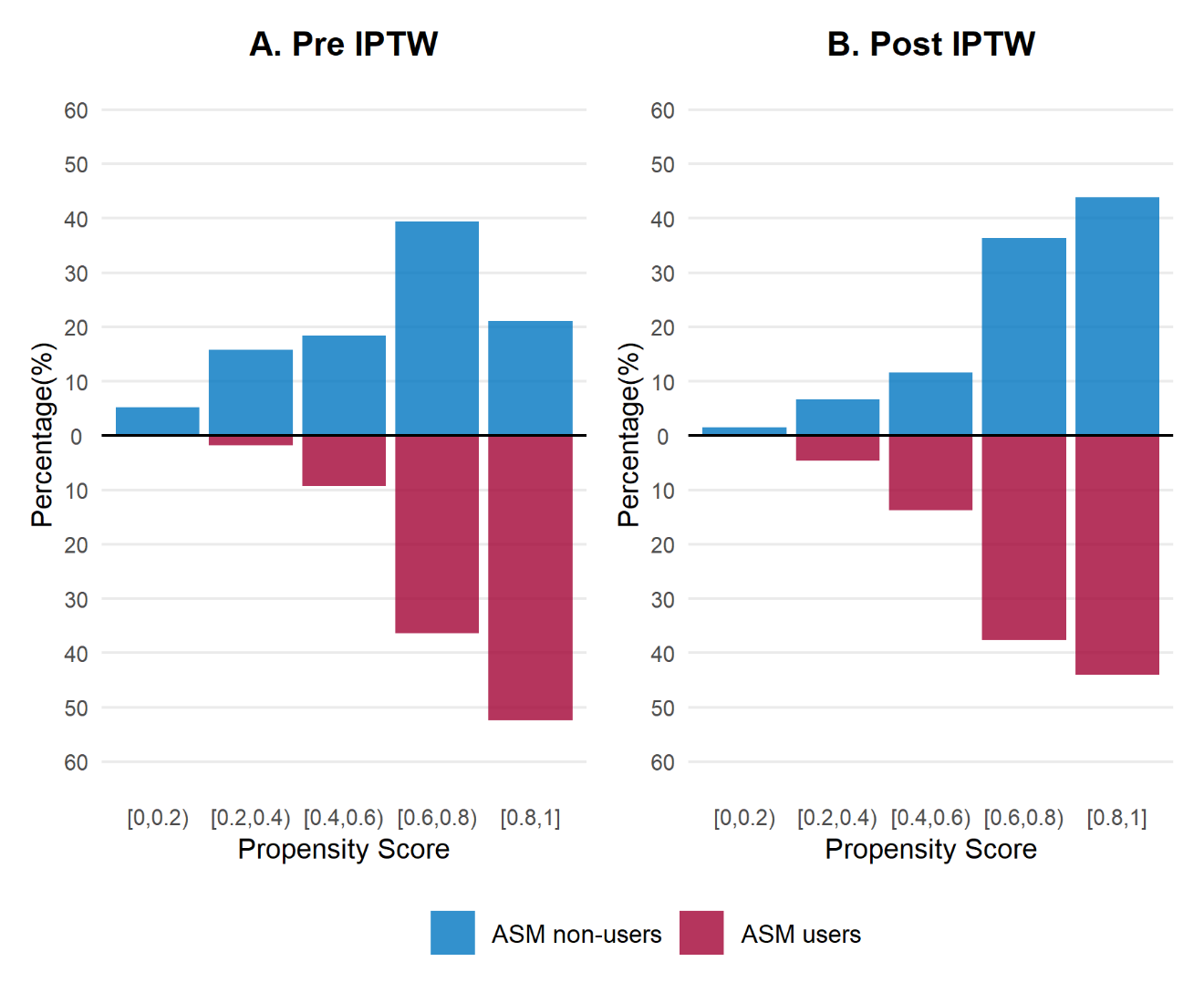


Figure A2. Standardized difference between ASMs users and ASMs non-users pre- and post- Inverse Probability of Treatment Weighting (IPTW).


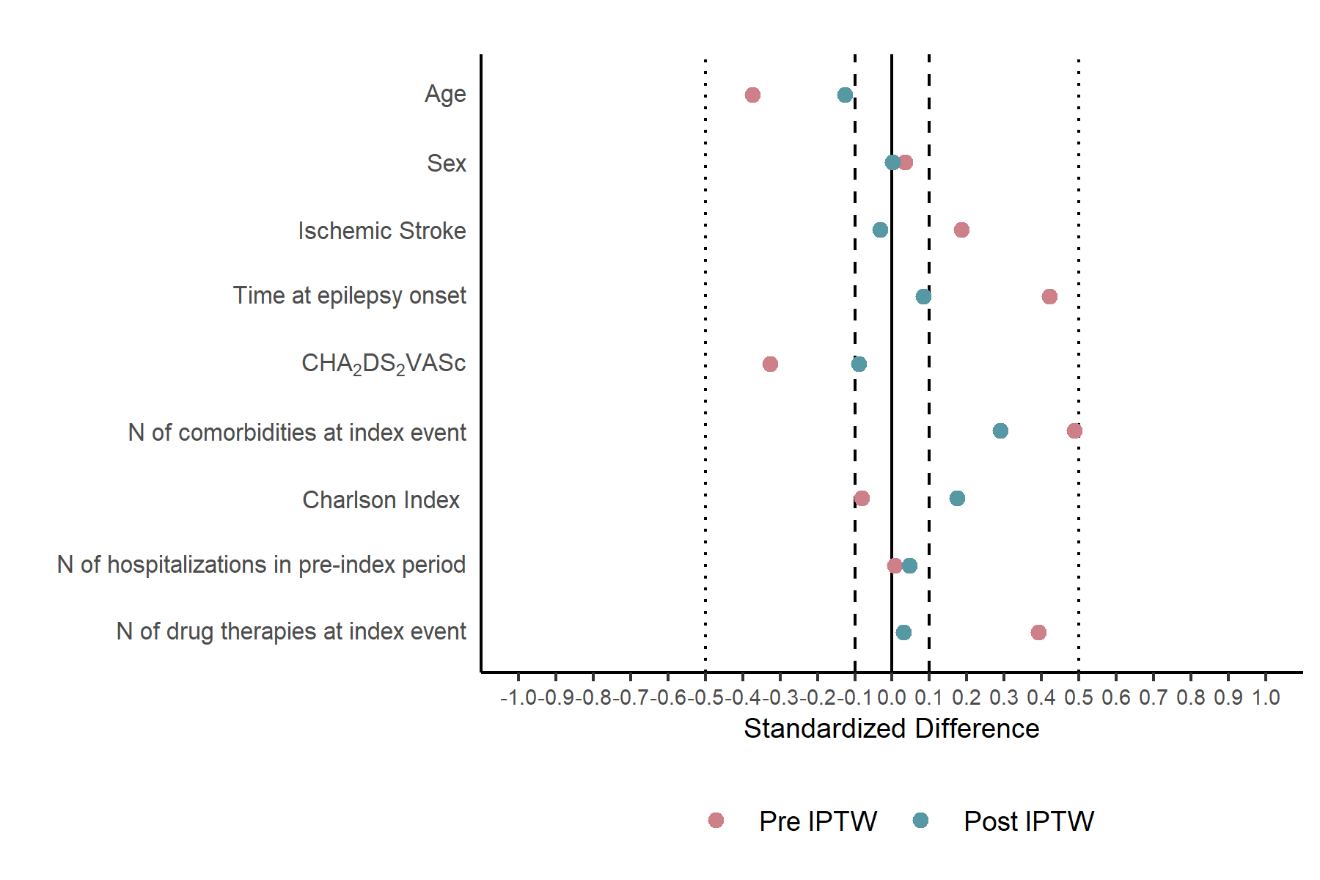

Supplement: Supplementary file 1 — Appendix S1. [file EPI-67-686-s001.docx]
